# Supplementary material for: Microenvironment-related gene TNFSF13B predicts poor prognosis in kidney renal clear cell carcinoma
Source: PeerJ. 2020 Jun 30;8:e9453. doi: 10.7717/peerj.9453 (PMC7333652; doi:10.7717/peerj.9453)
Supplement: Supplemental Information 1 [file peerj-08-9453-s001.zip › Supplemental Information/decription.docx]

1. Scores: The score of every sample
2. Score_survival: The input data of survival analysis
3. Score_stage: The input data of exploring relationship between score and stage
4. DEG: We use the score divide the samples into two groups and obtained differentially expressed genes (DEGs)
5. Interaction_Genes: We take the intersection of up-regulated genes, and so do down-regulated genes.
6. GO_KEGG: The result of GO and KEGG of interaction genes
7. MCODE_result: The result of MCODE.
8. Prognostic_gene_identified: The result of Differential expression, Kaplan-Meier and univariate Cox analysis, then we make intersection of them.
9. GSEA: The result of GSEA.
